# Therapeutic Efficacy of Metronomic Doxorubicin and Low Dose of Gamma Radiation on Breast Cancer Model

**DOI:** 10.1002/cbf.70278

**Published:** 2026-08-13

**Authors:** Ibrahim Y. Abdelrahman, Mostafa Saif‐Elnasr, Zeinab R. Attia, Rania A. Abd El Azeem, Eman Khalifa, Mostafa A. Askar

**Affiliations:** ^1^ Radiation Biology Department, National Center for Radiation Research and Technology Egyptian Atomic Energy Authority Cairo Egypt; ^2^ Health Radiation Research Department, National Center for Radiation Research and Technology Egyptian Atomic Energy Authority Cairo Egypt; ^3^ Children's Hospital, Faculty of Medicine Mansoura University Mansoura Egypt; ^4^ Medical Laboratories Department, Faculty of Applied Health Science Technology Nile Valley University Fayoum Egypt

**Keywords:** apoptosis, breast cancer, cell cycle, low dose of gamma radiation, metronomic dose of doxorubicin

## Abstract

Breast cancer is the most common malignancy in females internationally. Doxorubicin (DOX) has been well‐thought‐out as the most effective regimen for breast cancer treatment for several years. The chronic side effects of DOX obligate us to control the dosage that can be used, although its efficacy. Radiotherapy is a vigorous regimen in breast cancer treatment, but correlated side effects are determining factors for a successful recovery. Combined metronomic chemotherapy and radiotherapy, at low doses, can reduce the side effects of single‐modality treatments. We have studied the dose 1.8 mg/kg twice per week for 8 weeks of DOX singly or with 0.5 Gy fractionated doses of gamma radiation on inducing cell death, cell cycle arrest, and apoptosis, and also P53, B‐cell lymphoma‐2 (Bcl‐2), Bcl‐2‐associated X (Bax), and caspase‐3 gene expression in an animal breast cancer model. Treatment with DOX resulted in upregulation of apoptosis and downregulation of cell division at an accumulated dose of 0.5 Gy, as well as downregulation of Bcl‐2 gene expression and upregulation of p53, Bax, and caspase‐3 gene expression in animals bearing breast cancer. In addition, DOX in combination with radiation decreased the tumor size. A metronomic dose of DOX, when used with a low dose of gamma radiation, with the least side effects, could be a successful treatment for breast cancer in clinical trials.

## Significance Statement

In summary, this research concluded that a low dose of radiation (LDR) enhanced the antitumor effect of the metronomic dose of doxorubicin (M‐DOX). The results indicated that LDR might augment and accelerate M‐DOX‐induced apoptosis in breast cancer cells, potentially associated with the Ki67 gene. The M‐DOX and LDR combination therapy noticeably inhibits the proliferation of breast tumors induced in rats by dimethyl benz[*a*]anthracene carcinogenesis and concurrently considerably reduces the reported doxorubicin toxicity in the cardiac region; all of these results were validated by histopathology. Regarding clinical implications, this combination therapy may improve the safety and efficacy of breast cancer treatment, but dose optimization and timing are critical.

**Abbreviations**
ALT: alanine aminotransferase
ANOVA: analysis of variance
AST: aspartate aminotransferase
Bax: B‐cell lymphoma‐2‐associated X
Bcl‐2: B‐cell lymphoma‐2
BW: body weight
CK‐MB: creatine kinase MB
DMBA: dimethyl benz[*a*]anthracene
DOX: doxorubicin
EAEA: Egyptian Atomic Energy Authority
GSH: glutathione
HER2: human epidermal growth factor receptor 2
HR: hormone receptor
H&E: hematoxylin and eosin
LDR: low dose of radiation
MDA: malondialdehyde
M‐DOX: metronomic dose of doxorubicin
NCRRT: National Center for Radiation Research and Technology
NIH: National Institutes of Health
PBS: phosphate‐buffered saline
PI: propidium iodide
RT‐PCR: real‐time polymerase chain reaction
SEM: standard error of mean
SPSS: Statistical Package for the Social Sciences
TNBC: triple‐negative breast cancer

## 1 Introduction

Breast cancer is the main cause of death related to cancer among women. There are many subtypes of breast cancer that have been identified: a hormone receptor (HR)‐positive, human epidermal growth factor receptor 2 (HER2)–overexpressing, and triple‐negative breast cancer (TNBC) [1]. TNBC has a higher mortality rate than other types of breast cancer due to its high rate of recurrence [2, 3, 4]. The nontargeting anticancer drugs (paclitaxel, cyclophosphamide, and doxorubicin [DOX]) are used to treat breast cancer, especially in cases of TNBC where HR and HER2 are missed [5]. DOX is an anthracycline antibiotic and a broad‐spectrum anticancer agent [6, 7]. Although DOX is useful for the treatment of TNBC, its use is limited due to its severe side effects: cardiotoxicity, diarrhea, vomiting, hair loss, and nausea. The final‐accumulated dose of DOX must not exceed 450–500 mg/m^2^ because of its cardiotoxicity [6]. Also, another study used a low dose of DOX, two cycles of 20 mg/m^2^ versus five cycles of 30 mg/m^2^ as a standard of care [8]. Furthermore, reductions in DOX dosage to address its side effects reduce its therapeutic efficacy [9]. DOX is known to generate intracellular superoxide and hydrogen peroxide, which can mediate mitochondrial damage and apoptosis, and induce oxidative stress, leading to cytotoxicity in tumor cells [10].

Unlike treatment with high doses of radiation that causes considerable DNA damage resulting in injury and P53 activation, exposure of cells or whole animal bodies to low dose of radiation (LDR) can induce a protective radio‐adaptive response [11].

Despite ample information about the contribution of the p53 pathway to high doses of radiation‐induced effects [12]. The p53 protein (molecular policeman) [13] is a key regulator of the cell cycle and cell metabolism, inhibiting angiogenesis, apoptosis, and DNA repair [14]. Breast tumors that have elevated p53 are more regularly negative for estrogen receptor and are linked with a higher rate of proliferation and a lower rate of survival [13].

Therefore, after DOX treatment, p53 protein expression increases in response to DNA damage [13]. DOX dosage is limited due to the severe side effects, especially the cardiomyopathy incidence that leads to irreversible congestive heart failure [15]. The other most common side effects include: declined blood cell counts, elevated risks of infection and bleeding, loss of appetite, stomatitis, hair loss, nausea and vomiting, mouth sores, birth defects, liver toxicity, and acute arrhythmia [16].

Gamma rays are used to treat some forms of tumors. Gamma rays exert ionizing effects by transferring energy to irradiated cells, producing DNA damage, and thereby killing cancer cells, or by modulating the tumor stromal compartment, resulting from exposure to LDR [17, 18]. Cancer cells have varying levels of radio‐sensitization. In general, cells that grow more are more sensitive to radiation. Previous studies have shown that small doses of DOX, which exhibit the least cell toxicity, when used in combination with ionizing radiation in cancer cell lines, may be most effective for treating breast cancer [19, 20]. Farooqi et al. [21] recently conducted a promising Phase II clinical study, SATURN‐STS: Phase II study of neoadjuvant atezolizumab with DOX, concurrent atezolizumab with preoperative radiation therapy, and adjuvant atezolizumab in patients with high‐risk surgically resectable extremity and truncal soft tissue sarcoma. However, to the best of our knowledge, the effects of combination metronomic–chemoradiotherapy in breast cancer models in vivo and as a radio‐sensitization efficacy have not yet been evaluated. The side effects of chemotherapy will be significantly reduced when it is used at low doses, but its anticancer efficacy will be achieved when it is combined with another anticancer regimen, such as LDR. Therefore, this research article aims to explore the synergism between the metronomic dose of DOX (M‐DOX) and LDR in animal breast cancer models.

## 2 Materials and Methods

### 2.1 Drugs and Chemicals

Dimethyl benz[*a*]anthracene (DMBA) (≥ 95%) was obtained from Toronto Research Chemicals Inc., 20 Martin Ross Avenue, Toronto, Canada. DOX HCI injection USP, 200 mg/100 mL (2 mg/mL), Pfizer Pharm Company, was purchased from El‐Tarshouby Pharmacy, Mansoura, Egypt. DNase/RNase‐free water was obtained from Thermo Fisher Scientific Inc., USA. Acetone and all other used solvents were of high analytical grade and used as received.

### 2.2 Radiation Exposure

The animals were subjected to gamma irradiation using a Canadian Gamma Cell‐40 with a ^**137**^Cs source (Atomic Energy of Canada Ltd., Ontario, Canada) at the National Center for Radiation Research and Technology (NCRRT), Egyptian Atomic Energy Authority (EAEA), at a dose rate of 0.33 Gy/min. The animal's whole body was exposed to a 0.5‐Gy fractionated dose at 0.25 Gy every 10 days [17].

### 2.3 Animals

Female Sprague–Dawley rats weighing 50 ± 7 g (6–7 weeks of age) were obtained from the animal unit in NCRRT, EAEA. Rats were kept for acclimatization under standard laboratory conditions of controlled room temperature (25°C ± 1°C) on a 12 h light/dark cycle for about 2 weeks. They were allowed free access to water and food. All animal experiments were carried out in accordance with the ethical guidelines of the National Institutes of Health (NIH) concerning the care and use of laboratory animals (NIH Publication No. 85–23, revised 1985). The trials were approved by the NCRRT, EAEA Research Ethics Committee (No. F/23 A/24).

### 2.4 Breast Cancer Induction and Experimental Design

**Figure 1:**
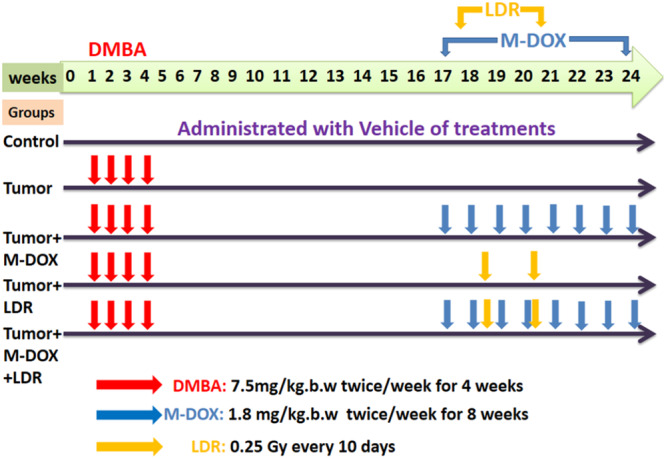
The experimental design and steps of different treatments. BW, body weight; DMBA, dimethyl benz[*a*]anthracene; LDR, low dose of radiation; M‐DOX, metronomic dose of doxorubicin.

The experiment was conducted for 24 weeks. After the adaptation period, the animals were divided into two experimental groups, as illustrated in Figure 1.

*Group A:* 10 female rats served as the normal control group and received no treatment.

Group B: 40 female rats that received DMBA (dissolved in 0.5 mL sesame oil and 0.5 mL saline) according to a previous study with some modification [22], at a dose of 7.5 mg/kg body weight (BW), two times a week for four consecutive weeks, intermammary glands. After 12 weeks from the last dose of DMBA, confirming the induction success of breast tumor in rats by the histopathological test. The tumor‐bearing animals were classified into four groups (eight rats/group) as follows:

*Group I:* Tumor‐bearing rats receive no treatment and serve as the positive control.
*Group II:* Tumor‐bearing rats injected with M‐DOX as the M‐DOX‐treated group, at a dose of 1.8 mg/kg BW two times/week for eight consecutive weeks [8].
*Group III:* Tumor‐bearing rats exposed to a low dose of gamma radiation as the LDR‐treated group; the animals' whole bodies were exposed to a 0.5‐Gy fractionated dose at 0.25 Gy every 10 days.
*Group IV:* Tumor‐bearing rats injected with M‐DOX as a combination group, at a dose of 1.8 mg/kg BW twice per week for eight consecutive weeks and exposed to a 0.5‐Gy fractionated dose, at 0.25 Gy every 10 days.

At the end of the experimental period, rats were given thiopental 50 mg/kg BW and atropine 50 µg/kg BW [23]. After all, blood was sampled, the animal died, and the tissue remains were buried in quicklime. Blood samples were collected from the heart and were placed in a red‐top tube for serum analysis of aspartate aminotransferase (AST), creatine kinase MB (CK‐MB), alanine aminotransferase (ALT) activities, glutathione (GSH) content, and malondialdehyde (MDA).

After removing the breast tissues, they were dissected into three parts; the first part was immediately immersed in liquid nitrogen for assessment of P53, caspase‐3, B‐cell lymphoma‐2 (Bcl‐2), and Bcl‐2‐associated X (Bax) relative messenger RNA (mRNA) expression by real‐time polymerase chain reaction (RT‐PCR). The second part was stored at −80°C after washing in phosphate‐buffered saline (PBS) to determine cell cycle, Annexin‐V, and Ki67 by flow cytometry technique. The third part was fixed in phosphate‐buffered formalin 10% (pH 7.2), processed, then embedded in paraffin for histopathological investigation.

### 2.5 Flow Cytometry Assay

Cell cycle analysis, Annexin‐V staining, and Ki67 staining were performed by flow cytometry. Briefly, after homogenization of 0.1 g of frozen breast tumor in PBS, the resulting suspension was washed twice with PBS. The cell pellets were resuspended after centrifugation, then incubated at room temperature for 30 min in 1 mL of propidium iodide (PI)/Triton X‐100 staining solution (0.1% Triton X‐100 in PBS, 0.2 mg/mL RNase A, and 10 mg/mL PI). For the apoptosis analysis, 100 μL of cell suspension was taken in another 5 mL test tube, then 5 μL of Annexin‐V kit reagent (Cat. No. 556419 BD) was added. PI (5 μL) (PE label) was added, and the tubes were incubated for 15 min in the dark at room temperature. After incubation, the cells were resuspended in 200 μL 1X binding buffer. For the cell surface markers Ki67, cells were stained with 10 μL monoclonal antibody (anti‐Ki67) (Biosciences, CA, USA) Cat. No. 566963 in the dark for 30 min at room temperature [24].

### 2.6 RT‐PCR

Total RNA extraction from mammary tissue of all groups was performed using the RNeasy Plus Mini Kit (Qiagen, CA, USA). The extracted RNA concentration and quality were measured using a spectrophotometric technique with a Nanophotometer (Implen GmbH, Germany). Reverse transcription of total RNA (1 μg) into single‐stranded complementary DNA (cDNA) was done using SensiFAST cDNA Synthesis Kit (Bioline, USA). The Primer 3 website was used to design specific PCR primers according to the sequence of nucleotide got from the National Gene Bank (Table 1). The designed primers and their oligonucleotide properties were analyzed using Net Primer (PREMIER Biosoft, USA). All primers were also blasted on the NCBI/BLAST website to confirm their specificity for the gene of interest. Rat GAPDH was used as a housekeeping gene and an internal reference control. RT‐PCR was achieved using Applied Biosystems Step One plus RT‐PCR (Thermo Fisher Scientific Inc., USA). Concisely, the template cDNA (2 μL) was amplified using a specific primer pair, 10 μL of 2x SensiFAST SYBR No‐ROX mix (Bioline, USA), and nuclease‐free water to a total reaction volume of 20 μL. Thermal cycling conditions were included in the initial activation step at 95°C for 2 min followed by 40 cycles of denaturation at 95°C for 5 s, annealing for 10 s, and extension at 72°C for 15 s followed by melting curve analysis. Relative expressions of rat P53, Bcl‐2, Bax, and caspase‐3 mRNA were calculated following the $2^{‐∆∆C_{T}}$ method.

**Table 1:** The primers used for amplification of the genes being studied.

Genes	Forward primers	Reverse primers
P53	ACAGCGTGGTGGTACCGTAT	GGAGCTGTTGCACATGTACT
Bcl‐2	GGATTGTGGCCTTCTTTGAGTTC	AGAGCGATGTTGTCCACCAG
Bax	ACAGGGTTTCATCCAGGATCGAG	AGCTCCATGTTGTTGTCCAGTTC
Caspase‐3	ACAGCGTGGTGGTACCGTAT	GGAGCTGTTGCACATGTACT
GAPDH	TGCCACTCAGAAGACTGTGG	TTCAGCTCTGGGATGACCTT

Abbreviations: Bax, B‐cell lymphoma‐2‐associated X; Bcl‐2, B‐cell lymphoma‐2; GAPDH, glyceraldehyde‐3‐phosphate dehydrogenase.

### 2.7 Colorimetric Assay

Serum GSH content and MDA levels were spectrophotometrically assessed by BioDiagnostic (Egypt) kits. A Labomed spectrophotometer (Los Angeles, USA) was used to measure the sample absorbance. Serum ALT and AST activities were determined kinetically using the Human Diagnostics (Germany) kit. Additionally, the activity of serum CK‐MB was measured using kinetic assay kits obtained from BioDiagnostic (Egypt).

### 2.8 Histopathological Examination of Breast and Heart Tissues

Mammary and heart tissues fixed in 10% formalin were embedded in paraffin, then cut into 5 μm thick sections, followed by staining with hematoxylin and eosin (H&E). A digital camera mounted on an Olympus BX51 optical microscope was used to photograph the histopathological alterations. Inflammation, fibrosis, necrosis, benign, and malignant were assessed in H&E‐stained mammary and heart tissues [25].

### 2.9 Statistical Analysis

Data were presented as mean ± standard error of mean (SEM). The results were evaluated statistically using one‐way analysis of variance (ANOVA), followed by Tukey's post hoc test. Cardiac and breast lesion histopathological scores did not fit a normal distribution; therefore, nonparametric comparisons were performed using the Kruskal–Wallis test, followed by Dunn's method. Statistical analysis was done using Statistical Package for the Social Sciences (SPSS) Version 20 (Chicago, IL, USA). The level of significance was fixed at *p* < 0.05. Graphing was done using GraphPad Prism software (GraphPad Software Inc., Version 8, San Diego, USA). Data are expressed as mean ± SEM, a^1^ *p* < 0.001, a^2^ *p* < 0.01, a^3^ *p* < 0.05 versus control group; b^1^ *p* < 0.001, b^2^ *p* < 0.01, b^3^ *p* < 0.05 versus tumor group; c^1^ *p* < 0.001, c^2^ *p* < 0.01, c^3^ *p* < 0.05 versus M‐DOX group; d^1^ *p* < 0.001, d^2^ *p* < 0.01, d^3^ *p* < 0.05 versus LDR group.

## 3 Results

### 3.1 Effect of M‐DOX and Radiotherapy on Cell Morphology

**Figure 2:**
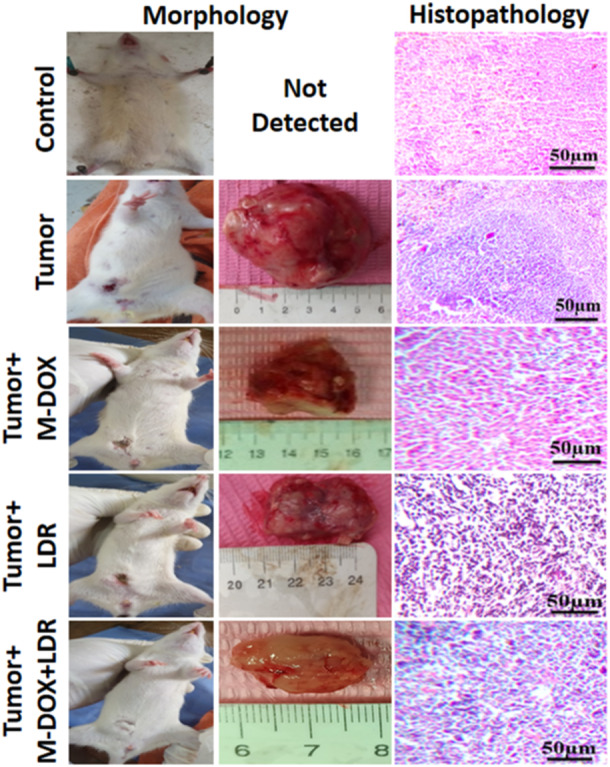
M‐DOX and/or LDR affect related to histopathological factors. (Normal control group) viewing healthy mammary acini embedded in the surrounding adipose tissue. Positive control (tumor group) displays several empty spaces in the cribriform adenocarcinoma. The top‐left image shows adenocarcinoma; notice the several spaces within the tumor's solid masses: some are empty, and others contain pink, proteinaceous secretion. (LDR group) present cancer cell necrosis, highlighted by large eosinophilic granule areas within the tumor masses. (M‐DOX group) viewing marked cancer cells necrosis with its transformation into hyalinized eosinophilic material, as well as many pyknotic nuclei. (M‐DOX + LDR group) display necrosis and the onset of fibrous bands among the groups of cancer cells. Tumor size was determined by a standard caliper, in width and length, upon excision of all untreated and treated tumor‐bearing rats. The section's microscopic examination was carried out by the Labomed Fluorescence microscope LX400, Cat No. 9126000; US and Labomed camera software, USA. LDR, low dose of radiation; M‐DOX, metronomic dose of doxorubicin.

**Figure 3:**
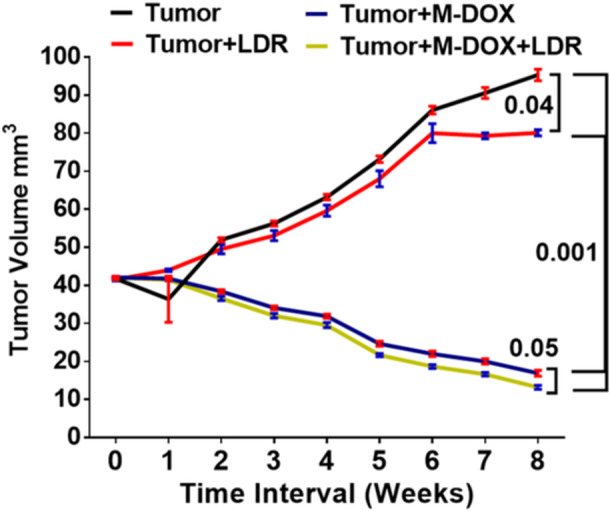
Showing the change in tumor volume (mm^3^) through 8 weeks in different groups. LDR, low dose of radiation; M‐DOX, metronomic dose of doxorubicin.

As shown in Table 2, 20% of mortality was noted in animal numbers after DMBA injection (intraperitoneal [*ip*]), which may be due to DMBA toxicity. The animals treated with M‐DOX and/or LDR showed a significant reduction in tumor volume in comparison with the positive control. Moreover, M‐DOX significantly enhanced the LDR effect, leading to a greater decrease in tumor volume compared with the single treatment (Figures 2 and 3).

**Table 2:** M‐DOX and/or LDR effect on survival percentage and tumor volume.

Groups			Control	Tumor	Tumor + M‐DOX	Tumor + LDR	Tumor + M‐DOX + LDR
Zero time	Animal numbers	*N*	10	8	8	8	8
	Tumor volume (mm^3^)	Mean	—	41.77	42.09	41.54	42.06
		SEM	—	0.34	0.24	0.38	0.24
	Life animals	Survival %	100	100	100	100	100
Week 1	Animal numbers	*N*	10	7	8	7	8
	Tumor volume (mm^3^)	Mean	—	36.36	41.77	43.95	41.47
		SEM	—	6.07	0.3	0.33	0.21
	Life animals	Survival %	100	87.5	100	87.5	100
Week 2	Animal numbers	*N*	10	6	7	6	7
	Tumor volume (mm^3^)	Mean	—	51.95	38.41 ^a1^	49.51 ^a^	36.54 ^a1^
		SEM	—	0.53	0.43	1.21	0.57
	Life animals	Survival %	100	75	88	75	87.5
Week 3	Animal numbers	*N*	10	5	7	5	7
	Tumor volume (mm^3^)	Mean	—	56.25	34.08 ^a1^	53.07 ^a^	32 ^a1^
		SEM	—	0.65	0.48	1.33	0.63
	Life animals	Survival %	100	63	87.5	63	87.5
Week 4	Animal numbers	*N*	10	5	7	5	7
	Tumor volume (mm^3^)	Mean	—	63.2	31.86 ^a1^	59.63 ^a^	29.55 ^a1^
		SEM	—	0.73	0.53	1.49	0.7
	Life animals	Survival %	100	63	87.5	63	87.5
Week 5	Animal numbers	*N*	10	4	6	4	6
	Tumor volume (mm^3^)	Mean	—	73.15	24.64 ^a1^	68.02 ^a1^	21.64 ^a1^
		SEM	—	0.86	0.63	2.11	0.44
	Life animals	Survival %	100	50	75	50	75
Week 6	Animal numbers	*N*	10	4	6	4	6
	Tumor volume (mm^3^)	Mean	—	86.06	21.98 ^a1^	80.02 ^a1^	18.65 ^a1^
		SEM	—	1.01	0.7	2.48	0.48
	Life animals	Survival %	100	50	75	50	75
Week 7	Animal numbers	*N*	10	3	6	4	6
	Tumor volume (mm^3^)	Mean	—	90.57	19.98 ^a1^	79.3 ^a1^	16.65 ^a1^
		SEM	—	1.45	0.7	0.77	0.48
	Life animals	Survival %	100	37.5	75	50	75
Week 8	Animal numbers	*N*	10	3	6	4	6
	Tumor volume (mm^3^)	Mean	—	95.33	16.88 ^a1^	80.1 ^a1^	13.18 ^a1^
		SEM	—	1.53	0.78	0.78	0.54
	Life animals	Survival %	100	37.5	75	50	75

*Note:* The results are presented as the mean ± SEM.

Abbreviations: LDR, low dose of radiation; M‐DOX, metronomic dose of doxorubicin; SEM, standard error of mean.

^a1^ *p* < 0.01, ^a^ *p* < 0.05 and > 0.01 versus Tumor group.

As shown in Figure 2, the control rats' breast tissue has no morphological changes, while the positive control group has a large breast tumor solid mass. A noticeable deterioration in tumor size was detected after M‐DOX or LDR treatment; a significant increase in this regression was achieved by the combination treatment of M‐DOX and LDR.

Our study showed that LDR can enhance the M‐DOX effect on breast cancer progression and attenuate its cardiac toxicity. We also observed the rat's general appearance, the animal survival rate, and changes in tumor volume. Different appearances were observed among different experimental groups, as shown in Figure 2. The animals in the positive control and LDR groups were 37.5% and 50% survived, respectively (Table 2), whereas rats in the M‐DOX group and M‐DOX + LDR showed 75% survival during the 8 weeks from treatment.

The histopathological examination of the mammary gland (Figure 2) confirmed that the control group exhibits numerous rounded secretory lobes with grape‐like structures, composed of epithelial cells, nestled within adipose tissue. Each lobe is characterized as a compound tubular acinar gland. The acini discharge into ducts lined with cuboidal or low columnar epithelial cells, encircled by myoepithelial cells. The ducts originating from each lobule are released into a lactiferous duct, encompassed by smooth muscle.

But in the tumor group, the mammary tissue exhibits disrupted architecture, with an irregular cell arrangement in contrast to the organized structure seen in normal breast tissue (Figure 2). A noticeable increase in cellularity is observed, accompanied by marked dysplastic changes, including nuclear atypia characterized by enlarged size, irregular shape, and increased chromatin content. Malignant cells exhibit elevated mitotic figures, signifying a high proliferation index. The stromal tissue surrounding breast cancer cells displays a desmoplastic reaction, exhibiting fibrous components and extensive collagen deposition.

Treated breast cancer tissue with M‐DOX exhibited a subtle decrease in tumor size, indicative of the inhibition of tumor cell proliferation. This inhibition was discernible through a reduction in mitotic activity and an improvement in the number of cells that divide rapidly. Furthermore, an increase in apoptosis was observed, marked by cell shrinkage and an elevated number of apoptotic bodies. Additionally, the detection of new blood vessels was evident in the treated tissue (Figure 2).

The mammary tissue of breast cancer exposed to LDR showed a mild reduction in tumor size, and disruption of tissue pattern, associated with changes in nuclear morphology and disaggregation of nuclear chromatin. In addition, fibrotic changes are characterized by an increased amount of collagen fibers. Infiltration of immune cells, such as lymphocytes and macrophages, into the tissue is well detected.

Breast cancer tissue subjected to a combination of M‐DOX + LDR exhibited a noticeable decrease in tumor size. This reduction was accompanied by a decline in cell proliferation and an increase in apoptosis (Figure 2). These alterations were linked to the amelioration of degenerative changes associated with DOX therapy, as evidenced by reduced fibrosis and decreased inflammatory infiltrates.

**Figure 4:**
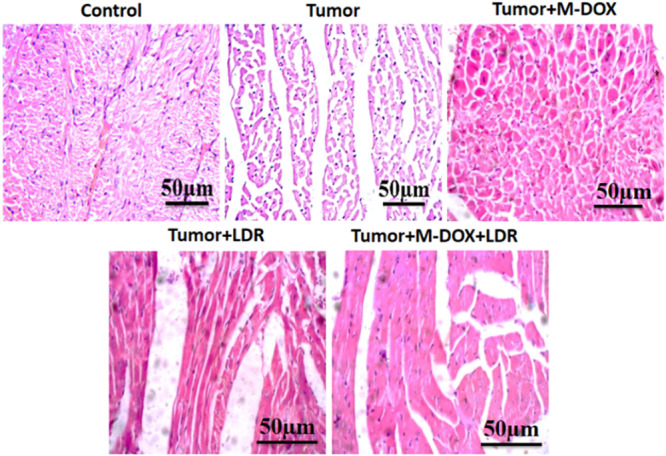
M‐DOX and/or LDR affect the histopathological features of heart tissue. The section's microscopic examination was carried out by the Labomed Fluorescence microscope LX400, Cat No. 9126000; US and Labomed camera software, USA. LDR, low dose of radiation; M‐DOX, metronomic dose of doxorubicin.

In the healthy group, the heart tissue exhibits normal histological features. The myocardium displays well‐organized cardiac muscle fibers with striations and intercalated discs (Figure 4). Cardiomyocytes show a regular arrangement, with centrally located nuclei. Blood vessels appear normal, and there is no evidence of inflammation or fibrosis. The overall architecture reflects the typical histological characteristics of healthy cardiac tissue.

In the breast cancer model group, the heart tissue exhibits histological changes associated with the presence of cancer. These changes include alterations in cardiac muscle fibers, increased interstitial fibrosis, and potential inflammatory infiltrates.

In the breast cancer model treated with M‐DOX, the cardiotoxic effects are evident without differences with tumor group. Histological features include disorganized cardiac muscle fibers, nuclear abnormalities, and signs of inflammation or fibrosis (Figure 4).

In the breast cancer model treated with LDR, the heart tissue did not show variations, with little improvement compared with the tumor‐positive group (Figure 4). The LDR exerts a protective effect, preserving cardiac histology from deterioration and reducing cancer‐induced changes.

In the breast cancer model treated with M‐DOX + LDR, histological examination reveals notable changes in the heart tissue. There is evidence of cardiotoxicity, characterized by disorganized cardiac muscle fibers, nuclear abnormalities in cardiomyocytes, and signs of inflammation. Additionally, interstitial fibrosis may be observed, but not to the extent observed in the tumor and M‐DOX groups. These histopathological changes reflect the mild impact of DOX on the myocardium, which was attenuated due to LDR exposure (Figure 4).

### 3.2 Cell Cycle Analysis by Flow Cytometry

**Figure 5:**
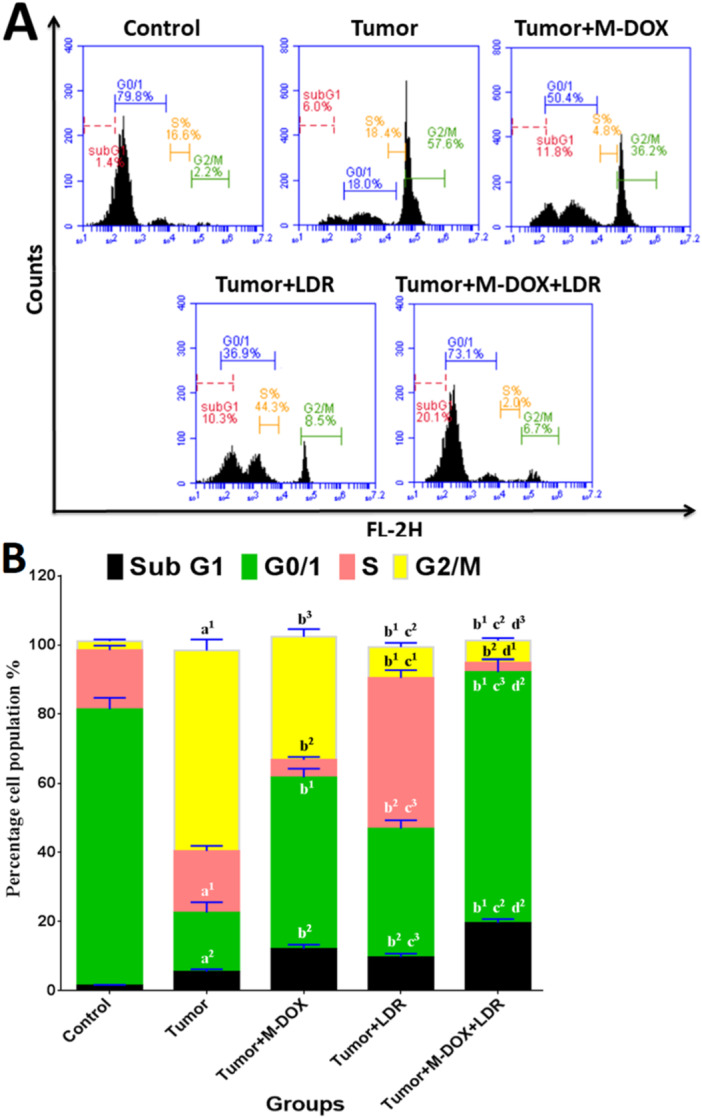
Effects of M‐DOX and LDR treatment on cell cycle analysis. Cell cycle histogram (A) and cell cycle chart (B) of the mammary tumor. Data are expressed as mean ± SEM, at a^1^ *p* < 0.001, a^2^ *p* < 0.01, a^3^ *p* < 0.05 versus control group; b^1^ *p* < 0.001, b^2^ *p* < 0.01, b^3^ *p* < 0.05 versus tumor group; c^1^ *p* < 0.001, c^2^ *p* < 0.01, c^3^ *p* < 0.05 versus M‐DOX group. d^1^ *p* < 0.001, d^2^ *p* < 0.01, d^3^ *p* < 0.05 versus LDR group. LDR, low dose of radiation; M‐DOX, metronomic dose of doxorubicin; SEM, standard error of mean.

We investigated the effects of M‐DOX and/or LDR on the distribution of the cell population in the breast cancer model. Animals treated with M‐DOX only showed a gradual increase of cells in the G1 phase compared with the tumor group (Figure 5A,B). An increase in the G1 phase was associated with a progressive increase of cells in sub‐G1 and a progressive decrease of cells in the S and G2/M phases compared with the tumor group.

The cell cycle underwent an arrest at the S phase because of radiation treatment, compared with the tumor and tumor + M‐DOX groups, associated with a progressive increase of cells in sub‐G1, compared with the tumor group, while the decrease of cells in sub‐G1 was observed compared with the tumor + M‐DOX group (Figure 5A,B). Also, we observed a progressive decrease in cells in the G1 and G2M phases, indicating that the synergetic factor stabilizes the treatment efficacy.

The results showed that combination treatment (M‐DOX + LDR) was significantly more effective than M‐DOX or radiation separately by arresting the cell cycle at the G1 phase and significantly increasing the proportion of the Sub‐G1 phase compared with all groups (Figure 5A,B). An increase in the Sub‐G1 and G1 phases was associated with a progressive decrease in cells in the S and G2M phases.

### 3.3 Apoptosis Analysis by Flow Cytometry

**Figure 6:**
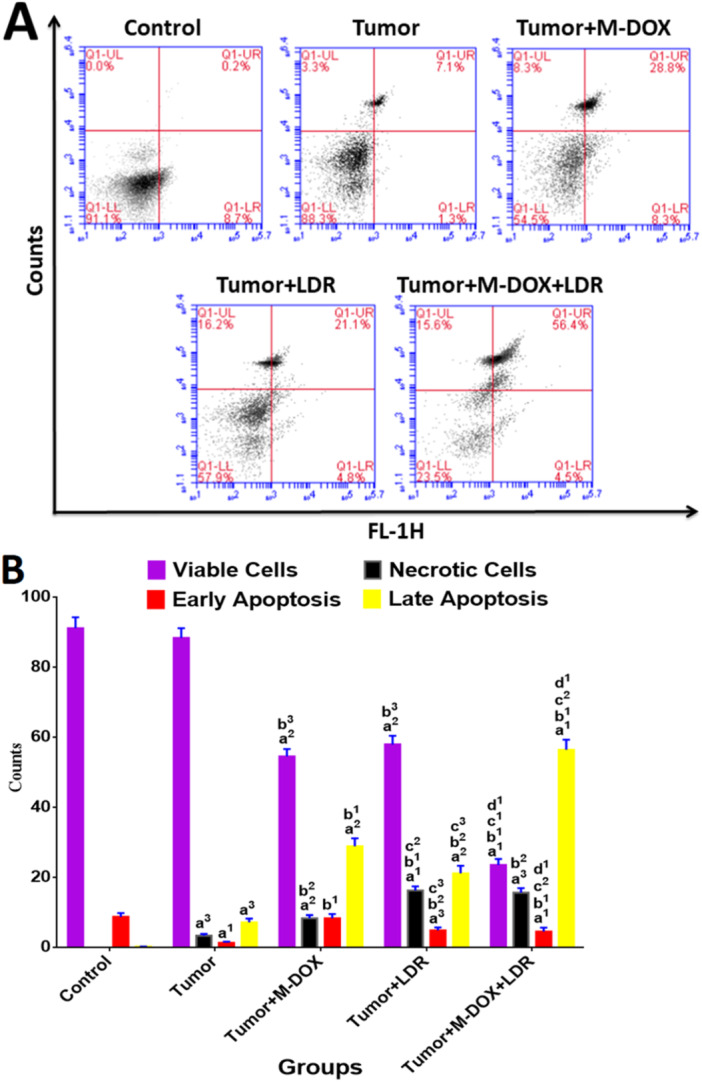
Effects of M‐DOX and LDR treatment on cell death. Apoptotic histogram (A) and apoptotic chart (B) of Annexin‐V positive cells were evaluated in mammary tumors. Results are expressed as % of positive cells from five independent experiments. Data are expressed as mean ± SEM, at a^1^ *p* < 0.001, a^2^ *p* < 0.01, a^3^ *p* < 0.05 versus control group; b^1^ *p* < 0.001, b^2^ *p* < 0.01, b^3^ *p* < 0.05 versus tumor group; c^1^ *p* < 0.001, c^2^ *p* < 0.01, c^3^ *p* < 0.05 versus M‐DOX group. d^1^ *p* < 0.001 versus the LDR group. LDR, low dose of radiation; M‐DOX, metronomic dose of doxorubicin; SEM, standard error of mean.

These data suggest that the inhibition of cell proliferation induced by M‐DOX and LDR can be due to the induction of apoptosis. To verify this possibility, mammary tissue in all groups was stained with Annexin‐V and analyzed by flow cytometry to detect viable, necrotic, and apoptotic cells. As shown in Figure 6A,B, significant differences were observed in the number of apoptotic cells; the percentage of apoptotic cells in the tumor group was not significantly different from that in the normal control group. The percentage of apoptotic cells induced by M‐DOX or LDR in the treated group was significantly higher than that of the tumor group (*p* < 0.001 and *p* < 0.01, respectively, vs. the tumor untreated group). Furthermore, the apoptotic cell count in the combined treatment group was significantly higher than that in the tumor group and that in M‐DOX or LDR alone (*p* < 0.001, 0.01, and 0.001, respectively). Meanwhile, the combination treatment was significantly more effective on tumor progression than M‐DOX or LDR groups alone.

### 3.4 Proliferation Marker Analysis by Flow Cytometry

**Figure 7:**
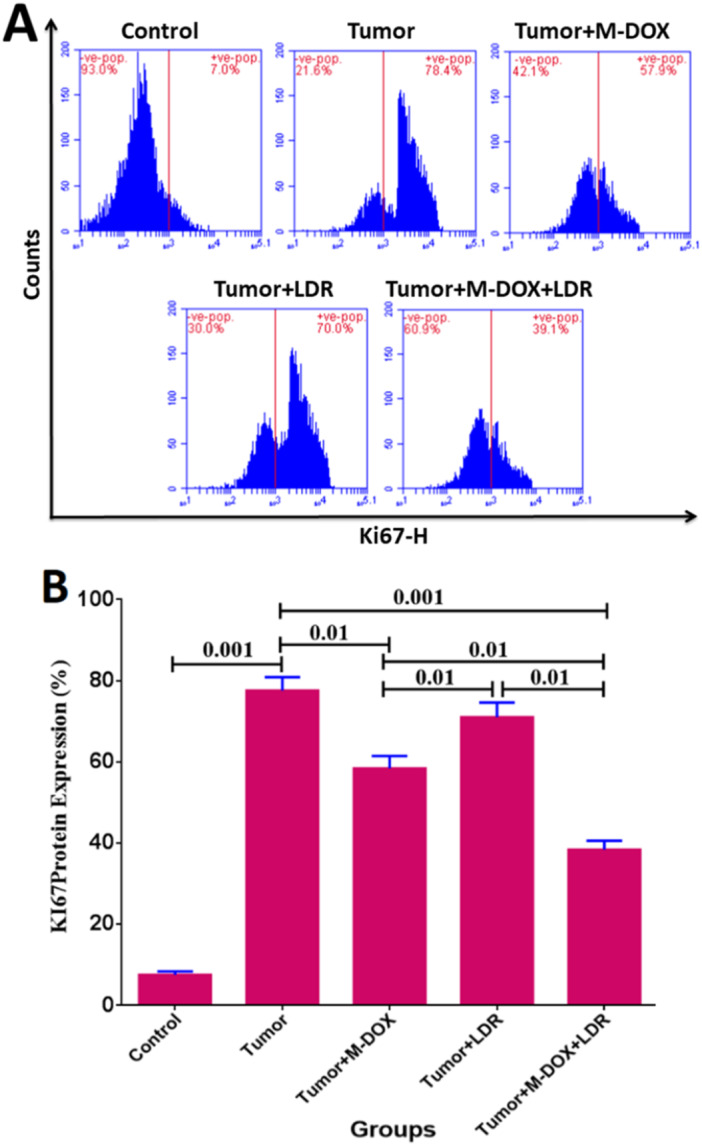
Gating scheme for flow cytometry analysis of intracellular Ki67 expression in mammary tissues. (A) Percentage of Ki67 in different treated groups. (B) Cumulative data showing frequencies of Ki67 cells in each group. Significance was determined by one‐way analysis of variance. Data are expressed as mean ± SEM, *p* < 0.001 and 0.01. LDR, low dose of radiation; M‐DOX, metronomic dose of doxorubicin; SEM, standard error of mean.

Quantitative analysis of Ki67‐stained cells showed that ~79% of cells in untreated tumor groups were positive for the cell proliferation marker Ki67. However, animals exposed to M‐DOX showed a significant suppression in Ki67 expression (~58%). However, the LDR group showed a reduction in Ki67 expression (~70%). In contrast, the combination of M‐DOX + LDR results in a profound decrease in the Ki67 expression (~39%), suggesting that the preemptive treatment with M‐DOX before LDR could hinder the regressive proliferation activity in mammary tumors (Figure 7).

### 3.5 Apoptosis Analysis by RT‐PCR

DOX, as a potent chemotherapy drug, is widely used in the treatment of breast cancer through intercalating into DNA and inhibiting topoisomerase, leading to DNA damage and apoptosis. The expression of apoptosis‐related proteins such as P53, Bax, Bcl‐2, and Caspase‐3 is crucial for understanding the molecular mechanisms underlying M‐DOX‐induced cell death in cancer cells.

**Figure 8:**
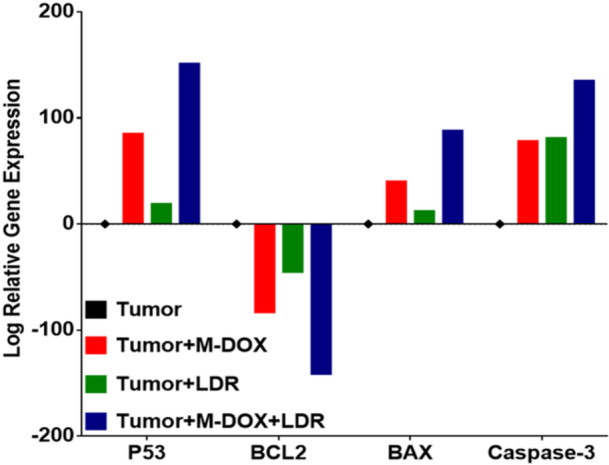
The change in gene expression of the regulatory P53 gene, in addition to the down‐regulation of antiapoptotic gene Bcl‐2, and the up‐regulation of apoptotic gene expression, including Bax and caspase‐3 genes. Data are expressed as relative gene expression. Bax, B‐cell lymphoma‐2‐associated X; Bcl‐2, B‐cell lymphoma‐2; LDR, low dose of radiation; M‐DOX, metronomic dose of doxorubicin.

In the current study, the results are valid for the activation of the P53 tumor suppressor, a critical mediator of cellular response to DNA damage, and its activation triggers a cascade of events that lead to cell cycle arrest via P53‐dependent transcription of P21 or apoptosis via P53‐dependent transcription of proapoptotic genes like Bax and caspase‐3 and the antiapoptotic Bcl‐2 gene. The P53 gene expression showed fold changes of 7.15, 1.54, and 32.5 in M‐DOX, LDR, and combination therapy (M‐DOX and LDR), respectively. But the Bcl‐2 gene expression recorded fold of change 0.148, 0.351, and 0.039 in M‐DOX, LDR, and combination therapy, respectively, as mentioned in Table 3, and illustrated in Figure 8. Our results were followed by upregulation of the Bax gene, with 2.5‐, 1.3‐, and 7.5‐fold changes, and caspase‐3 showed 5.96‐, 6.5‐, and 22.6‐fold changes in M‐DOX, LDR, and combination therapy, respectively. The results showed that M‐DOX, singly or in combination with LDR, orchestrates a complex interplay in gene expression among P53, Bax, Bcl‐2, and caspase‐3. The M‐DOX combined with LDR achieved significantly greater results than M‐DOX alone or LDR alone and effectively shifted the balance between proapoptotic and antiapoptotic signals, promoting apoptosis in breast cancer in rat models and enhancing treatment outcomes. Understanding these molecular mechanisms is essential for optimizing therapeutic strategies and developing more effective breast cancer treatment protocols.

**Table 3:** Shows the relative gene expression of P53, Bcl‐2, Bax, and caspase‐3 in different groups under study.

	**Parameters**											
	P53 relative gene expression			Bax relative gene expression			Bcl‐2 relative gene expression			Caspase‐3 relative gene expression		
Groups	CT	RQ	Log RQ (%)	CT	RQ	Log RQ (%)	CT	RQ	Log RQ (%)	CT	RQ	Log RQ (%)
Tumor	30.0	1	0	29.4	1	0	24.3	1	0	32.9	1	0
Tumor + M‐DOX	24.9	7.2	85	25.9	2.5	40	24.9	0.1	−83	28.1	6.0	78
Tumor + LDR	29.5	1.5	19	29.1	1.3	12	25.9	0.4	−45	30.3	6.5	81
Tumor + M‐DOX + LDR	27.0	32.5	151	28.5	7.5	88	31.0	0.0	−141	30.4	22.6	135

Abbreviations: Bax, B‐cell lymphoma‐2‐associated X; Bcl‐2, B‐cell lymphoma‐2; CT, cycle threshold; LDR, low dose of radiation; M‐DOX, metronomic dose of doxorubicin; RQ, relative quantification.

### 3.6 Serum Biomarkers

**Figure 9:**
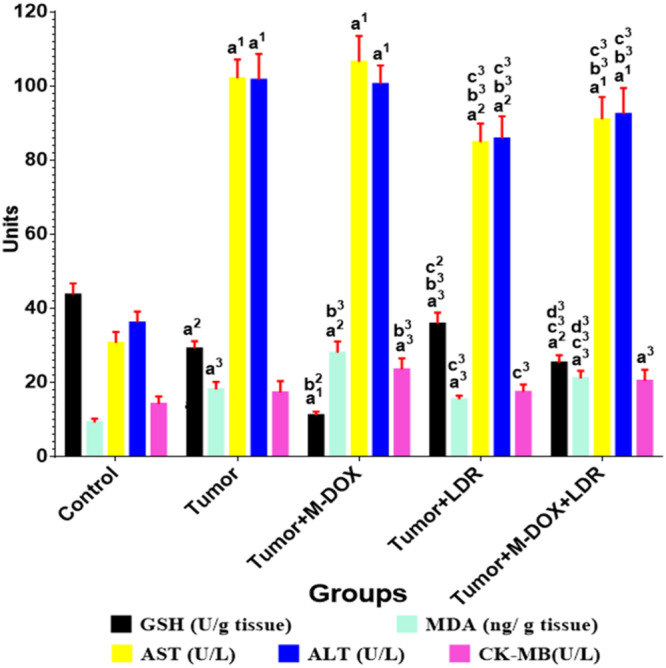
The change in the serum biomarkers for antioxidant (GSH), oxidative stress (MDA), liver (AST and ALT) and cardiac (CK‐MB) functions. Data are expressed as mean ± SEM, at a^1^ *p* < 0.001, a^2^ *p* < 0.01, a^3^ *p* < 0.05 versus control group; b^2^ *p* < 0.01, b^3^ *p* < 0.05 versus tumor group; c^2^ *p* < 0.01, c^3^ *p* < 0.05 versus M‐DOX group; d^3^ *p* < 0.05 versus the LDR group. ALT, alanine aminotransferase; AST, aspartate aminotransferase; CK‐MB, creatine kinase MB; GSH, glutathione; LDR, low dose of radiation; MDA, malondialdehyde; M‐DOX, metronomic dose of doxorubicin; SEM, standard error of mean.

To explore the systematic protective effect of LDR against M‐DOX, slight side effects, antioxidant and oxidative stress markers in serum were assessed. The results showed that GSH significantly decreased and MDA increased in the tumor group compared with the normal group. Also, compared with the tumor group, the concentration of GSH was significantly decreased, and MDA was significantly increased in the serum of the M‐DOX group, whereas GSH increased significantly and MDA decreased in the LDR group. As well, compared with the sole M‐DOX group, GSH was significantly increased, and MDA was significantly decreased in the combination M‐DOX with LDR group (Table 4 and Figure 9).

**Table 4:** The serum biomarkers for antioxidant (GSH), oxidative stress (MDA), liver and cardiac functions.

	**Parameters**				
**Groups**	**GSH (U/g tissue)**	**MDA (ng/g tissue)**	**AST (U/L)**	**ALT (U/L)**	**CK‐MB (U/L)**
Control	43.8 ± 3	9.3 ± 1	30.7 ± 3	36.2 ± 3	14.3 ± 2
Tumor	29.2 ± 2 ^a^ ^2^	18.2 ± 2 ^a^ ^3^	102.2 ± 5 ^a^ ^1^	101.7 ± 7 ^a^ ^1^	17.4 ± 3
Tumor + M‐DOX	11.2 ± 1 ^a^ ^1,^ ^b^ ^2^	28.1 ± 3 ^a^ ^2,^ ^b^ ^3^	106.6 ± 7 ^a^ ^1^	100.6 ± 5 ^a^ ^1^	23.6 ± 3 ^a^ ^3,^ ^b^ ^3^
Tumor + LDR	35.9 ± 3 ^a^ ^3,^ ^b^ ^3,^ ^c^ ^2^	15.5 ± 1 ^a^ ^3,^ ^c^ ^3^	84.9 ± 5 ^a^ ^2,^ ^b^ ^3,^ ^c^ ^3^	85.9 ± 6 ^a^ ^2,^ ^b^ ^3,^ ^c^ ^3^	17.5 ± 2 ^c^ ^3^
Tumor + M‐DOX + LDR	25.4 ± 2 ^a^ ^2,^ ^c^ ^3,^ ^d^ ^3^	21.2 ± 2 ^a^ ^3,^ ^c^ ^3,^ ^d^ ^3^	91.1 ± 6 ^a^ ^1,^ ^b^ ^3,^ ^c^ ^3^	92.5 ± 7 ^a^ ^1,^ ^b^ ^3,^ ^c^ ^3^	20.5 ± 3 ^a^ ^3^

*Note:* The results are presented as the mean ± SEM. ^a^ ^1^ *p* < 0.001, ^a^ ^2^ *p* < 0.01, ^a^ ^3^ *p* < 0.05 versus control group; ^b^ ^2^ *p* < 0.01, ^b^ ^3^ *p* < 0.05 versus tumor group; ^c^ ^2^ *p* < 0.01, ^c^ ^3^ *p* < 0.05 versus M‐DOX group; ^d^ ^3^ *p* < 0.05 versus the LDR group.

Abbreviations: ALT, alanine aminotransferase; AST, aspartate aminotransferase; CK‐MB, creatine kinase MB; GSH, glutathione; LDR, low dose of radiation; MDA, malondialdehyde; M‐DOX, metronomic dose of doxorubicin.

Cardiovascular and hepatic toxicities accompany the efficiency of DOX treatment, so the results at this point focused on serum biomarkers of liver and cardiac functions, specifically ALT, AST, and CK‐MB. According to the current findings, DMBA tumor formation significantly increased liver function in the positive control compared with the normal control. By using M‐DOX, there is no significant difference in ALT or AST compared with the positive control; however, in case of LDR and combination with M‐DOX combination, there is a significant decrease compared with the positive control.

Regarding CK‐MB as a cardiac‐specific isoenzyme that serves as a sensitive marker for myocardial inflammation or injury, there is a non‐significant difference compared with the normal control due to tumor induction by DMBA. While M‐DOX use demonstrated a slight adverse effect on cardiac function, where it caused a slight significant increase in CK‐MB activity. On the other hand, LDR did not differ from the positive control group, while it improved the slightly adverse effect of M‐DOX on CK‐MB activity when used in combination, as shown in Table 4 and Figure 9.

## 4 Discussion

Despite all efforts regarding breast cancer treatment at the level of academic and clinical status, the complete recovery rate of these cancers is not high enough and has not achieved the international target of curability. Among the diversity of chemotherapeutic regimens, DOX is one of the most commonly used anticancer agents, but its use is limited by its cytotoxicity, especially in patients with certain risk factors. Therefore, some changes in DOX doses will be needed to achieve the desired efficacy with low toxicity, so the new issue of metronomic dosing is applied in this study to use low doses over the long term. A previous study demonstrated that changes in organ weight were often due to DOX side effects [26]. Likewise, in mice, a consensus article confirmed the DOX negative impact on BW gain [20]. However, an adaptive protective response could be induced by pretreatment with LDR [27], thereby protecting normal tissue from DOX. Yu et al. [28] stated that LDR could stimulate the growth of normal cells but not that of tumor cells in vivo and in vitro. They also indicated that pretreatment of sarcoma‐bearing mice (S180) with whole‐body X‐rays (75 mGy) noticeably improved the antitumor effect of cyclophosphamide in reducing tumor growth [29]. Low‐dose radiation likewise prevented cancer metastasis and decreased self‐renewal ability and sphere formation in breast cancer [30].

In the current study, M‐DOX, when administered to breast tumor‐bearing rats with whole‐body gamma‐rays at a 0.5‐Gy fractionated dose, enhanced the antitumor effect. Although we did not have direct evidence of reduced tumor progression, we demonstrated M‐DOX's improvment impact on LDR‐induced suppression of proliferation‐related protein expression. Previous clinical phase II study indicated that concurrent DOX and intensity modulated radiation therapy appears to be effective in controlling Local Recurrence (LR disease) in patients with recurrent or respectable differentiated thyroid cancer [31]. Regarding the proliferation of tumors, the expression of Ki67, a nuclear proliferation marker, is an important cell proliferation index in breast cancer, malignant pleural mesothelioma, and prostate cancer [20]. Also, we showed here that M‐DOX or LDR could suppress tumor growth by decreasing Ki67‐positive cells; M‐DOX with LDR demonstrated a more significant reduction in Ki67‐positive cells in the tumor tissue, which was in good agreement with the changes in morphology. These findings suggested that LDR improved the M‐DOX cytotoxicity in breast cancers. One of the mechanisms through which LDR suppresses the growth of tumors may be related to its antitumor immune function that may increase natural killer activity, antibody formation, secretion of interferon, and other cytokines, which all can inhibit the growth of tumors, which, in fact, also negatively regulate tumor cell cycle and apoptosis by inhibiting cancer progression‐associated proteins [32].

Proliferation and invasion of tumors were complex processes mediated by several proteins, such as cell cycle regulators, p53, Bcl‐2, Bax, caspase‐3, and Annexin‐V. Bcl‐2 expression promotes the formation of pathological, invasive vessels throughout the process of neovascularization [33]. The existing study showed that treatment with M‐DOX, followed by LDR, further decreased, compared with M‐DOX or LDR alone, the expression of Bcl‐2‐induced cell cycle arrest through enhancement of apoptotic mediators. These findings suggest that M‐DOX and LDR together may be more effective in inhibiting tumor growth than either treatment separately.

The activation of apoptotic processes, or their regulation, by chemotherapy or radiotherapy in tumor cells plays a vital role in the antitumor effect. Here, the M‐DOX and LDR combination therapy noticeably promoted apoptosis. It was stated that radiotherapy increased tumor cell apoptosis by lowering Bcl‐2 gene expression, an antiapoptotic gene, in S180 sarcoma‐bearing mice [34], leading to mitochondria‐related caspase 3 cleavage‐mediated apoptosis [35, 36]. Our study showed that M‐DOX combined with LDR promoted tumor tissue apoptosis by elevating caspase‐3 and Bax and decreasing Bcl‐2, supporting the involvement of the mitochondrial apoptosis pathway. What is more, the preceding study has shown that M‐DOX with LDR had fewer adverse effects on cardiac tissue than the chemotherapeutic DOX standard, which is used in the treatment of cancer patients by suppressing oxidative stress and apoptosis [37]. The current research further confirms that M‐DOX with LDR may cause less cardiac damage while treating breast tumors by having substantial antitumor effects without causing adverse effects on cardiac tissue.

The present study demonstrated that LDR with M‐DOX decreased MDA levels and increased GSH antioxidant levels in serum compared with the M‐DOX‐treated group, indicating a protective effect of LDR against the side effects of M‐DOX. The decrease in MDA and increase in antioxidants demonstrated that lipid peroxidation was reduced and that the degree of cell damage was alleviated. Superoxide dismutase, catalase, and GSH are the main participants in the antioxidant defense system, which removes harmful oxidants [38]. Yu et al. [39] stated that LDR before chemotherapy induces antioxidant enzymes activities, promotes free radical scavenging, and decreases damage to liver tissues caused by high‐dose of cyclophosphamide. We found that LDR activated the antioxidant signaling pathway when used in combination with M‐DOX. Therefore, we hypothesized that LDR can activate the body's defense against the adverse effects of M‐DOX by upregulating antioxidants expression, thereby modulating vital organs functions, such as the liver and heart, as indicated by improvements in ALT, AST, and CK‐MB activities. Thus, LDR helps cells resist oxidative stress damage induced by tumor or hazardous chemotherapy. The toxicity of cardiac tissue due to DOX treatment may be linked to intracellular ROS generation, and antioxidants might reduce this toxicity without hindering the cytotoxic effect on lymphoid malignant tissue [40].

## 5 Conclusion

In summary, this research concluded that LDR improved M‐DOX antitumor effect. The results indicated that LDR might augment and accelerate M‐DOX‐induced apoptosis in breast cancer cells, potentially associated with the Ki67 gene. The M‐DOX and LDR combination therapy noticeably inhibits the proliferation of breast tumors induced in rats by DMBA carcinogenesis and concurrently considerably reduces the reported toxicity of DOX in the cardiac region, and all of these results were validated by histopathology. Regarding clinical implications, this combination therapy may improve the safety and efficacy of breast cancer treatment, but dose optimization and timing are critical.

## Author Contributions

**Ibrahim Y. Abdelrahman, Mostafa Saif‐Elnasr, Zeinab R. Attia, Rania A. Abd El Azeem, Eman Khalifa, Mostafa A. Askar:** conceptualization, data curation, formal analysis, validation, investigation, visualization, methodology, writing – review, and editing. All authors have read and agreed to the published version of the manuscript.

## Ethics Statement

Research Ethics Committee of EAEA, No. F/23A/24.

## Consent

The authors have nothing to report.

## Conflicts of Interest

The authors declare no conflicts of interest.

## Data Availability

All data generated or analyzed during this study are included in this manuscript.
